# Enhancement of 3-hydroxypropionic acid production from glycerol by using a metabolic toggle switch

**DOI:** 10.1186/s12934-015-0342-1

**Published:** 2015-10-05

**Authors:** Keigo Tsuruno, Hiroshi Honjo, Taizo Hanai

**Affiliations:** Laboratory for Bioinformatics, Graduate School of Systems Life Sciences, Kyushu University, 804 Westwing, 3-1-1 Maidashi, Higashi-ku, Fukuoka, 812-8582 Japan

**Keywords:** 3-Hydroxypropionic acid, *Escherichia coli*, Metabolic toggle switch, Synthetic pathway, Glycerol, Synthetic genetic circuit

## Abstract

**Background:**

3-hydroxypropionic acid (3-HP) is an important platform for the production of C3 chemicals, including acrylic acid, methyl acrylate, and acrylamide. Microbial production of 3-HP is mainly due to glycerol metabolism. In this study, in order to improve microbial 3-HP production, we applied a metabolic toggle switch for controlling the glycerol metabolism to redirect the excess metabolic flux of central metabolic pathway toward an exogenous 3-HP producing pathway in *Escherichia coli*.

**Results:**

The metabolic toggle switch enables conditional repression of the expression of a target gene during the fermentation. We individually performed conditional repression of *glpK*, *tpiA*, and *gapA,* which are involved in glycerol metabolism. The conditional repression of *glpK* and *tpiA* was not effective for 3-HP production under our experimental conditions. However, *gapA* conditional repression contributed to improve 3-HP production (titer, 54.2 ± 1.5 mM; yield, 32.1 ± 1.3 %) compared with that for the wild type strain. Additional deletion of endogenous *yqhD*, which is responsible for the production of a major byproduct, 1,3-propandiol, further increased 3-HP production (titer, 67.3 ± 2.1 mM; yield, 51.5 ± 3.2 %). The titer and yield were 80 and 94 % higher than those of the wild type strain, respectively. The obtained 3-HP yield from glycerol is comparable with the highest yield ever reported for microbial 3-HP production using glycerol as a sole carbon source. The measurement of intracellular metabolites showed the metabolic toggle switch successfully controlled the metabolic flux.

**Conclusion:**

The conditional repression of *gapA* by using the metabolic toggle switch combined with deletion of endogeneous *yqhD* increased 3-HP production approximately twofold from glycerol. This result indicates the metabolic toggle switch can be applied in various bio-production using diverse substrates.

## Background

Growing concerns regarding the depletion of fossil resources and environmental sustainability have led to an increased demand for the development of bio-based chemicals and fuel production using renewable feedstock by microorganisms. Recently, in several cases, an exogenous synthetic pathway introduced in non-native hosts sufficiently produced various alcohols, acids, and chemicals [1–6]. *Escherichia coli*, in particular, has mainly been used as the host organism for bio-production using this synthetic pathway, due to its well-understood metabolism and potential for easy genetic manipulation [7]. In many studies, conventional metabolic engineering, such as gene deletion which defects genes on chromosome responsible for competing pathway by homologous recombination, was applied to increase metabolic flux toward the synthetic pathway for improving the titer and yield of the final products [1, 8]. In many cases, the introduced synthetic pathway for carbon-based products such as alcohols or fatty acids competes with central carbon metabolism processes, including glycolysis and the tricarboxylic acid (TCA) cycle, for carbon sources. However, the deletion approach is unsuitable for enzymes that catalyze central carbon metabolism, as the deletion leads to growth defects or severely decreased growth of the host strain under certain conditions [9]. Therefore, conventional metabolic engineering strategies may not be feasible in the case of the deletion of such genes for improvement of bio-production. There is therefore a need for other strategies that increase the metabolic flux to the objective products rather than that to a central carbon metabolite, thus avoiding the growth defect during production.

To overcome this issue, there have recently been several attempts to control metabolic flux by modulating the expression levels of enzymes involved in the central metabolic pathway [10–13]. We previously developed a metabolic toggle switch (MTS) to control the metabolic flux by using a genetic toggle switch that functions as a genetic circuit composed of two repressor proteins and two repressible promoters in *E. coli* [14, 15]. For isopropanol production, we performed conditional repression of citrate synthase (EC 2.3.3.1) encoded by *gltA*, which catalyzes the condensation reaction of one molecule of acetyl-CoA and one molecule of oxaloacetate to one molecule of citrate, in the middle of fermentation by addition of isopropyl β-D-1-thiogalactopyranoside (IPTG). The conditional repression of *gltA* inhibited the metabolic flux from glycolysis to the TCA cycle and resulted in accumulation of acetyl-CoA. This acetyl-CoA accumulation resulted in increased titer and yield of isopropanol (derived from acetyl-CoA) and avoided the severe growth defect observed using the conventional gene deletion [15]. This encouraged the use of MTS for other bio-production processes employing a synthetic pathway to improve productivity. So far, studies controlling metabolic flux have been mainly focused on glucose metabolism. However, there has been no report of controlling the metabolic flux using substrates other than glucose, such as xylose or glycerol.

3-Hydroxypropionic acid (3-HP) is a three carbon non-chiral carboxylic acid that is often used as a platform for the production of several kinds of commercially important compounds, such as acrylic acid, methyl acrylate, acrylamide, ethyl 3-HP, malonic acid, propiolactone, and acrylonitrile [16]. Because of its usefulness, 3-HP was selected by the US Department of Energy as one of the top value-added chemicals produced with biomass [17]. 3-HP is produced through a two-step reaction from glycerol as follows: the first step is the conversion of glycerol to 3-hydroxypropionaldehyde (3-HPA), catalyzed by vitamin B_12_–dependent glycerol dehydratase, and the second step is the conversion of 3-HPA to 3-HP, catalyzed by aldehyde dehydrogenase. Recently, the growing demand for the production of biodiesel has led to a reduction in the price of crude glycerol, since glycerol is a major byproduct of biodiesel production [18, 19]. Therefore, glycerol has been regarded as an attractive carbon feedstock for 3-HP production.

To date, efforts to increase microbial 3-HP production have been made using engineered *E. coli* with an introduced synthetic pathway as a host organism. Mohan et al. optimized the fermentation conditions such as the pH, working volume, and initial glycerol concentration [20]. Rathnasingh et al. adjusted the expression level of enzymes in the synthetic pathway and demonstrated that the alternative enzyme exhibited higher activity [21]. Several recent studies focused on increasing the metabolic flux from glycerol toward 3-HP by the deletion of enzymes involved in glycerol metabolism [22, 23]. However, the deletion of several genes resulted in poor cell growth [22] and some of the deleted strains required glucose coupled with glycerol as an additional substrate for cell growth [23]. Thus, an alternative approach to improve 3-HP production that avoids severe growth defect without using an additional substrate other than glycerol is required.

Under aerobic conditions, glycerol is converted to glyceraldehyde 3-phosphate, one of the intermediates of glycolysis, through glycerol-3-phosphate and dihydroxyacetone phosphate. Each reaction in the pathway is catalyzed by four enzymes: glycerol kinase, glycerol-3-phosphate dehydrogenase, triosephosphate isomerase, and glyceraldehyde-3-phosphate dehydrogenase encoded by *glpK* (EC 2.7.1.30), *glpD* (EC 1.1.5.3), *tpiA* (EC 5.3.1.1), and *gapA* (EC 1.2.1.12), respectively. Thus, we speculated that the conditional repression of these enzymes engaged in glycerol metabolism could direct the excess metabolic flux of the central metabolic pathway toward the introduced synthetic pathway for 3-HP production.

Here, we applied a MTS that involved conditional repression of *glpK*, *tpiA* or *gapA* and a synthetic metabolic pathway for 3-HP production in order to increase the titer and yield of 3-HP by redirecting metabolic flux from the central metabolic pathway toward 3-HP production. Conditional repression of *gapA* effectively increased 3-HP productivity, and prevented severe growth inhibition. Moreover, additional deletion of *yqhD*, which catalyzes the reaction for producing a major byproduct of 1,3-propanediol (1,3-PDO), further increased the titer and yield of 3-HP.

## Results and discussion

### 3-HP production from glycerol via the synthetic metabolic pathway in *E. coli*

**Fig. 1:**
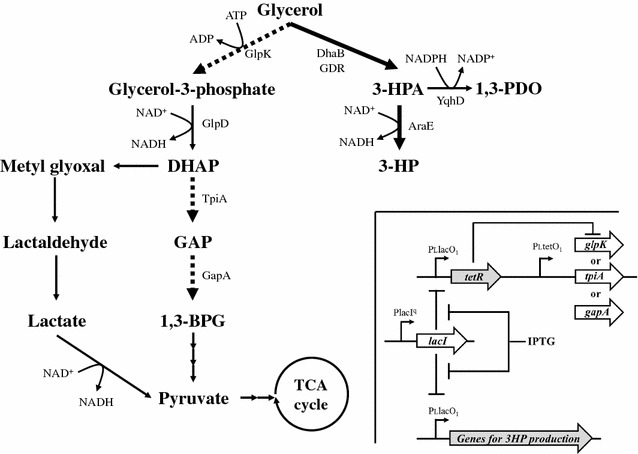
Metabolic pathway involved in central metabolism and 3-HP production from glycerol. *Dashed arrows* represent conditionally repressed reactions. *Bold arrows* represent the introduced synthetic pathway for 3-HP production. The *inset* indicates the structure of MTS. When IPTG was absent, LacI repressed the transcription from PllacO1. In the presence of IPTG, TetR repressed the transcription from PltetO1. Metabolite abbreviations: *DHAP* dihydroxyacetone phosphate, *GAP* glyceraldehyde 3-phosphate, *1,3-BPG* 1,3-bisphosphoglycerate, *3-HPA* 3-hydroxypropionaldehyde, *3-HP* 3-hydroxypropinic acid, *1,3-PDO* 1,3-propanediol. Metabolic enzymes: *GlpK* glycerol kinase, *GlpD* glycerol-3-phosphate dehydrogenases, *TpiA* triosephosphate isomerase, *GapA* glyceraldehyde-3-phosphate dehydrogenase, *DhaB* glycerol dehydratase, *GDR* glycerol dehydratase reactivator, *AraE* α-ketoglutaric semialdehyde dehydrogenase, *YqhD* 1,3-propandiol oxidoreductase

**Fig. 2:**
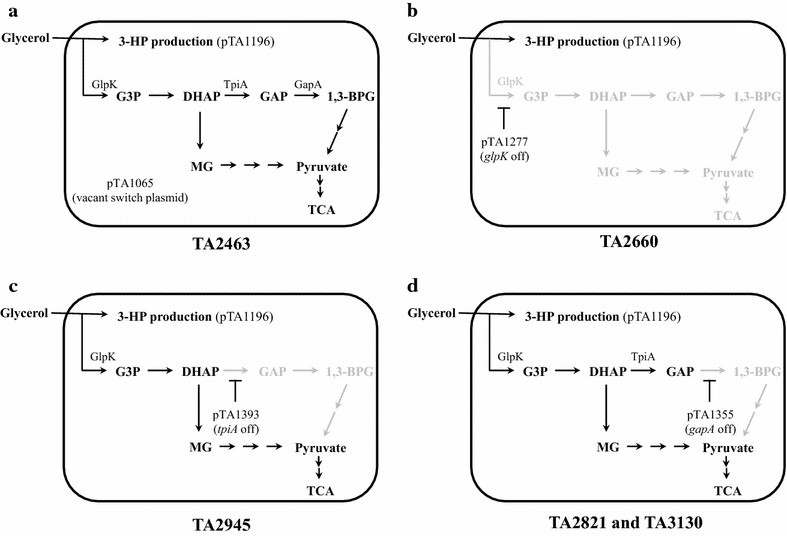
Illustrations of the strains used in this study. **a** TA2463, **b** TA2660, **c** TA2945 and **d** TA2821 and TA3130. The metabolites and reactions that would be decreased after conditional repression are indicated by *gray characters* and *arrows*. Metabolite abbreviations: *G3P* glycerol-3-phosphate, *MG* metyl glyoxal

TA2463, an *E. coli* strain based on TA1015 containing a plasmid pTA1196 encoding glycerol dehydratase (*dhaB*) and glycerol dehydratase reactivase (*gdrAB*) derived from *Klebsiella pneumoniae*, catalyze the conversion of glycerol to 3-HPA. α-ketoglutaric semialdehyde dehydrogenase (*araE*) derived from *Azospirillum brasilense* is also encoded on the plasmid that catalyzes the conversion of 3-HPA to 3-HP (Figs. 1, 2; Table 1). The expression of these enzymes for 3-HP production was induced by the addition of 0.1 mM IPTG at 0 h.

**Table 1:** Bacterial strains and plasmids used in this study

Strains/plasmid	Relevant characteristics	References/source
*E. coli* strains		
BW25113	lacIq rrnBT14 *ΔlacZ*WJ16 *hsdR*514 *ΔaraBAD*AH33 *Δrha*BADLD78	
JW0336	BW25113 *ΔlacI*::Kan^r^	[28]
JW3897	BW25113 *ΔglpK*::Kan^r^	[28]
JW3890	BW25113 *ΔtpiA*::Kan^r^	[28]
JW2978	BW25113 *ΔyqhD*::Kan^r^	[28]
TA1015	JW0336 removed Kan^r^	This study
TA2463	TA1015/pTA216, pTA1065, pTA1196	This study
TA2207	TA1015 *ΔglpK*	This study
TA2660	TA2207/pTA216, pTA1196, pTA1277	This study
TA2791	TA1015 *ΔtpiA*	This study
TA2945	TA2791/pTA216, pTA1196, pTA1393	This study
TA367	BW25113 *ΔgapA*, Kan^r^	Gifted by Dr. Nakahigashi
TA386	TA367 removed Kan^r^	This study
TA2732	TA386/pTA216, pTA1335	This study
TA2793	TA2732 *ΔlacI*/pTA216, pTA1335, Kan^r^	This study
TA2814	TA2793 removed Kan^r^	This study
TA2821	TA2814/pTA1196	This study
TA3125	TA2814 *ΔyqhD*/pTA216, pTA1335	This study
TA3130	TA3125/pTA1196	This study
Plasmids		
pCP20	Amp^r^, Cm^r^, FLP^+^, λcI857^+^, Rep^ts^	[29]
pTA216	pSC101*, Cm^r^, P_lacI_^q^::*lacI*	[15]
pTA867	ColE1, Kan^r^, PllacO_1_::*dhaB1*, *dhaB2*, *dhaB3*, *gdrA*, *gdrB*, *araE*	[24]
pTA958	p15A, Kan^r^, PllacO_1_::*tetR*, PltetO_1_::*gapA.*LAA	This study
pTA1065	p15A, Spec^r^, PllacO_1_::*tetR*, PltetO_1_::MCS	This study
pTA1196	ColE1, Kan^r^, PllacO_1_::*dhaB1*, *dhaB2*, *dhaB3*, *gdrA*, *gdrB* PllacO_1_::*araE*	This study
pTA1277	p15A, Spec^r^, PllacO_1_::*tetR*, PltetO_1_::*gltA*.LAA	This study
pTA1393	p15A, Spec^r^, PllacO_1_::*tetR*, PltetO_1_::*tpiA*.LAA	This study
pTA1355	p15A, Spec^r^, PllacO_1_::*tetR*, PltetO_1_::*gapA*.LAA	This study

**Fig. 3:**
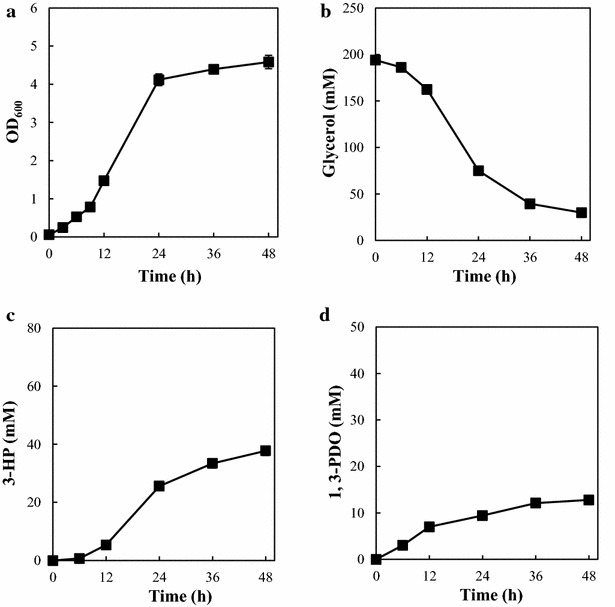
3-HP production by an *E. coli* strain harboring the synthetic pathway (TA2463). **a** Growth curve (OD_600_). **b** Time course of glycerol consumption. **c** Time course of 3-HP production. **d** Time course of 1,3-PDO production. *Error bars* represent the standard deviation (n = 3)

TA2463 produced 37.7 ± 1.6 mM 3-HP and the OD_600_ and glycerol consumption at 48 h were 4.58 ± 0.18, 163.9 ± 1.8 mM, respectively (Fig. 3a–c). The growth rate and 3-HP production decreased after 24 h, although glycerol remained in the medium. This was also observed in a previous study, in which 200 mM glycerol was used as a substrate for 3-HP production in batch culture [20]. The 3-HP yield from glycerol was 26.5 ± 1.1 % (mol/mol). The 3-HP production and yield observed was slightly lower than that previously reported in a batch culture study using the same enzymes for 3-HP production and similar culture conditions, including the use of a shake flask [21]. This could be due to the different protein expression systems used, which resulted in different protein expression levels. A significant amount of 1,3-propanediol (1,3-PDO) (12.8 ± 1.8 mM) (Fig. 3d), which is converted from 3-HPA in a reaction catalyzed by endogenous NADPH-dependent aldehyde reductase encoded by *yqhD*, was produced as the main byproduct, which is in agreement with previous studies [32–34]. Acetate has been shown to be a major byproduct of 3-HP production from glycerol in other studies [20–23]. In contrast, only small amounts of acetate (maximum of 2.18 ± 0.86 mM) accumulated at 24 h. Acetate was eventually removed, perhaps as a result of reassimilation in our experimental conditions. This observation corresponds to several reports performing fermentation of *E. coli* under aerobic conditions using minimal medium with glycerol as the sole carbon source [35–37]. Other metabolites such as lactate were not detected during fermentation. IPTG addition at various time points (3, 6, and 9 h) did not significantly affect growth, glycerol consumption, or 3-HP and 1,3-PDO production (data not shown).

### Effect of the conditional repression of *glpK* on 3-HP production

Glycerol kinase (*glpK*) catalyzes the conversion of glycerol to glycerol-3-phosphate, which enters glycolysis via dihydroxyacetone phosphate (DHAP) (Fig. 1). Deletion of *glpk* should result in increased yield of 3-HP from glycerol, but the deletion caused growth defects in *E. coli* cultured in M9 medium containing glycerol as the sole carbon source [23]. We therefore speculated that conditional repression of *glpK* using MTS was an appropriate strategy for achieving a high titer and yield of 3-HP, and would reduce the metabolic flux towards the central metabolic pathway.

**Fig. 4:**
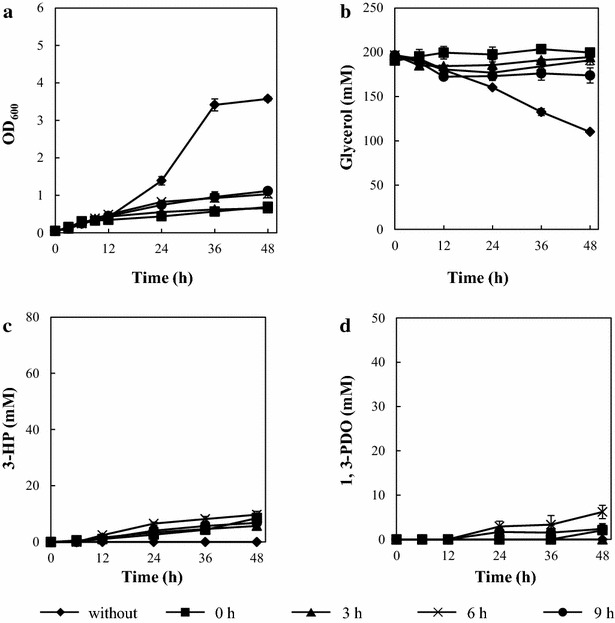
3-HP production by a *glpK* conditional repression strain (TA2660). **a** Growth Curve (OD_600_). **b** Time course of glycerol consumption. **c** Time course of 3-HP production. **d** Time course of 1,3-PDO production. *Error bars* represent the standard deviation (n = 3)

TA2660, a *glpK* conditional repression strain with the synthetic pathway for 3-HP, was constructed and subjected to 3-HP production (Fig. 2; Table 1). The conditional repression and expression of the introduced synthetic pathway were simultaneously induced by addition of IPTG [15]. To assess the effect of the timing of the conditional repression at various growth phases, IPTG was added to cultures at 0, 3, 6, and 9 h (from here on referred to as strain name_0, 3, 6, or 9 h). Uninduced TA2660 demonstrated reduced growth rate and decreased glycerol consumption compared to the wild type strain (TA2463) (Figs. 3a, b, 4a, b). Induction with IPTG at every time point tested resulted in severe growth defects and low glycerol consumption (Fig. 4a, b). Less than 10 mM of 3-HP and 1,3-PDO was produced (Fig. 4c, d). TA2660_0 h scarcely consumed glycerol through fermentation, while TA2660_9 h assimilated about 25 mM of glycerol within 12 h. However, glycerol assimilation stopped and 3-HP was produced at low levels after 12 h. Previously, Jung et al. showed that a *glpK*-deleted strain heterologously expressing *glpK*, controlled by the l-arabinose-inducible promoter, increased the 3-HP titer and yield from glycerol, whereas even in the absence of l-arabinose, the strain grew and produced 3-HP [23]. This suggests that our conditional repression strategy resulted in lower *glpK* activity than the l-arabinose dependent regulation method and significantly reduced intracellular metabolism. It was reported that the *glpK* deletion strain was able to efficiently grow and produce 3-HP using both glycerol and glucose substrates. In this strain, energy and cellular components were supplied from central carbon metabolism of glucose [33]. These results indicate that some amount of metabolic flux towards a central metabolic pathway such as glycolysis and the TCA cycle is required during 3-HP production to supply energy and cellular components.

### Effect of *tpiA* conditional repression on 3-HP production

In order to distribute a considerable amount of metabolic flux toward the central metabolic pathway, 3-HP production was initiated using TA2945, a 3-HP producing synthetic pathway and MTS strain with *tpiA* conditional repression (Figs. 1, 2; Table 1). Glycerol-3-phosphate dehydrogenase (GlpD) encoded by *glpD* is responsible for the conversion of glycerol-3-phosohate to DHAP (Fig. 1). Although this reaction is the immediate downstream of the reaction catalyzed by glycerol kinase (*glpK*), there is no metabolic pathway that catalyzes the glycerol-3-phosphate toward central metabolic pathway except for the reaction responsible for GlpD. We speculated that the conditional repression of *glpD* could not be expected a remarkable improvement of 3-HP production compared with the case of *glpK*. On the other hand, triosephosphate isomerase encoded by *tpiA* catalyzes the conversion of DHAP to glyceraldehyde-3-phosphate (GAP), such that the flux dispensed from DHAP can be converted to pyruvate by the methylglyoxal pathway via methylglyoxal, lactaldehyde, and lactate (Fig. 1). This methylglyoxal pathway is normally inactive [38]; however, high concentrations of DHAP can induce its activity [39].

**Fig. 5:**
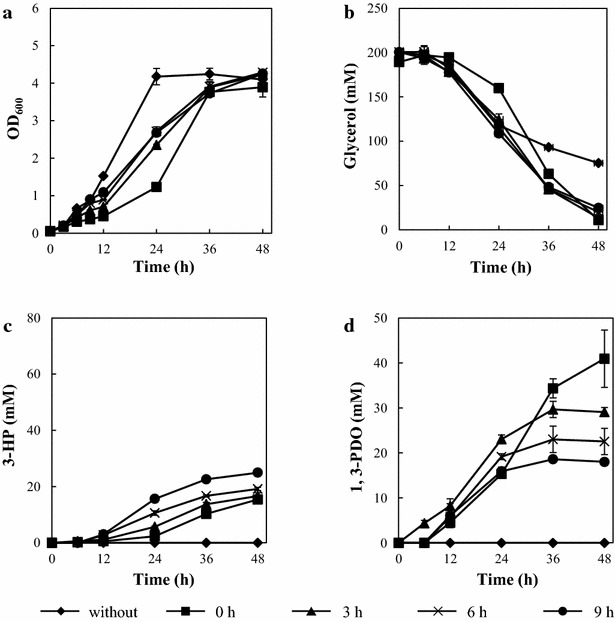
3-HP production by a *tpiA* conditional repression strain (TA2945). **a** Growth curve (OD_600_). **b** Time course of glycerol consumption. **c** Time course of 3-HP production. **d** Time course of 1,3-PDO production. *Error bars* represent the standard deviation (n = 3)

The growth profile of TA2945 without IPTG induction was similar to that of wild type strain (TA2463) (Figs. 3a, 5a). TA2945 strains induced with IPTG, especially TA2945_0 h, demonstrated lower growth rates than uninduced TA2945 strains until 24 h; however, the addition of IPTG did not affect the final OD_600_. In contrast to TA2660, TA2945 with IPTG, including TA2945_0 h, continued to assimilate glycerol after the addition of IPTG. Specifically, their glycerol consumption rate after 24 h was higher than that of TA2463 and consumed almost all of the glycerol in the medium at 48 h (Fig. 5b). In our previous report, the glucose consumption rate of the *gltA* conditional repression strain also surpassed that of the wild type strain, which did not have any particular genetic modifications [15]. It has been considered that the faster substrate consumption is attributed to the energy shortage of the cell [40]. TA2945 produced lactate (a maximum of 6.2 ± 0.23 mM produced by TA2945_3 h at 36 h), an intermediate of the methylglyoxal pathway. These results suggest that *tpiA* conditional repression led to the accumulation of DHAP, and that activation of the methylglyoxal pathway resulted in some amount of metabolic flux directed to the central metabolic pathway. Taking into account the decreased growth rate of TA2945 with IPTG induction, the metabolic flux toward the central metabolic pathway should be reduced compared to TA2463. However, IPTG-induced TA2945 produced the lowest titer and yield of 3-HP (Fig. 5c). TA2945_9 h produced the highest 3-HP titer and yield among the TA2945 induced at different time points, which were only 25.0 ± 0.75 mM and 14.2 ± 0.44 %, respectively. The titer and yield were 34 and 47 % lower than, respectively, in TA2463, indicating that conditional repression of *tpiA* is not effective for 3-HP production. Tokuyama et al. demonstrated 3-HP production from glycerol using an *E. colitpiA*-deficient strain [22]. The deletion of *tpiA* improved 3-HP titer and yield compared to the parental stain. However, the deletion led to significant growth retardation and decreased glycerol consumption, where it took about 100 h to reach the stationary phase [22]. This distinction demonstrates the difference in the effects between conditional repression and deletion of *tpiA* on metabolism.

Interestingly, TA2945 produced higher concentrations of 1,3-PDO (Fig. 5d). Particularly, 3-HP and 1,3-PDO concentration achieved by TA2945_0 h were 15.4 ± 1.4 and 40.9 ± 6.4 mM, respectively, showing that a large proportion of 3-HPA was not converted to 3-HP but was instead converted to 1,3-PDO (Fig. 1). This is similar to the results of a previous report on 3-HP production using a *tpiA* deletion strain [22]. NAD^+^ is required for both the conversion of 3-HPA to 3-HP and of lactate to pyruvate in the methylglyoxal pathway (Fig. 1). In addition, the reduced metabolic flux toward the central metabolic pathway would prevent NAD^+^ generation. Accordingly, the intracellular level of NAD^+^ would be insufficient for 3-HP production resulting in increased metabolic flux from 3-HPA to 1,3-PDO.

### Effect of *gapA* conditional repression on 3-HP production

To further evaluate the benefit of conditional repression for higher 3-HP production with distribution of some amount of metabolic flux to the central metabolic pathway, TA2821, a strain with *gapA* conditional repression due to an MTS having the 3-HP producing synthetic pathway, was constructed and tested for 3-HP production (Fig. 2; Table 1). Glyceraldehyde-3-phosphate dehydrogenase (GAPDH) encoded by *gapA* is responsible for the conversion of GAP to 1,3-bisphosphoglycerate (1,3-BPG) (Fig. 1).

**Fig. 6:**
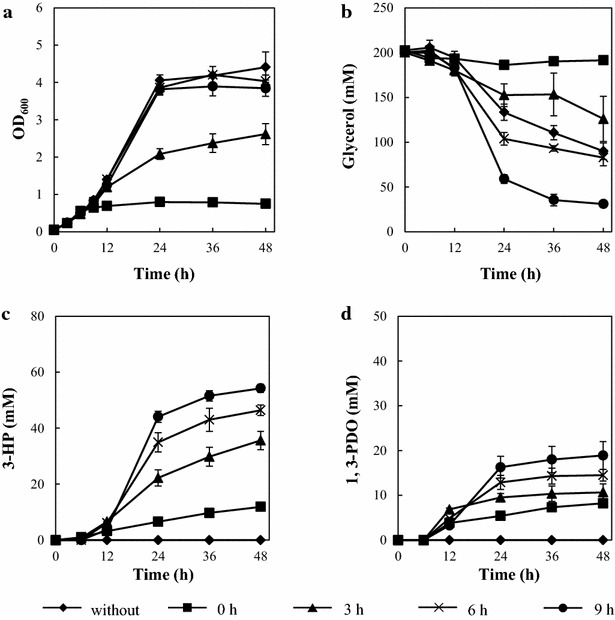
3-HP production by a *gapA* conditional repression strain (TA2821). **a** Growth curve (OD_600_). **b** Time course of glycerol consumption. **c** Time course of 3-HP production. **d** Time course of 1,3-PDO production. *Error bars* represent the standard deviation (n = 3)

The conditional repression of *gapA* initiated at 0 and 3 h resulted in growth inhibition that affected not only growth rate but also the final OD_600_. However, in the absence of IPTG, TA2821_6 and 9 h showed similar growth profiles that were comparable to the growth of wild type strain TA2463 (Figs. 3a, 6a). These results indicate that expression of *gapA* from a medium copy plasmid for 6 h was enough to induce energy and the necessary cell components for adequate cell growth. The glycerol consumption of TA2821_3 and 6 h were similar to the uninduced IPTG strain, but TA2821_9 h, demonstrated increased glycerol consumption (Fig. 6b). Importantly, TA2821_6 and 9 h produced higher concentrations of 3-HP, 46.4 ± 1.9 and 54.2 ± 1.5 mM, respectively. These concentrations represented a 23.0 and 43.9 % increase, respectively, compared to the 3-HP titer produced by TA2463. Furthermore, the yields of these conditions were 44.6 ± 4.2 and 32.1 ± 1.3 %, respectively. They were also higher than that of TA2463. These results indicate that conditional repression of *gapA* at 6 h and 9 h contributed to an increase in both 3-HP titer and yield from glycerol without impairing cell growth. This might be due to excess metabolic flux resulting from *gapA* repression, which provided improved 3-HP production.

In contrast to the results observed with the TA2945 *tpiA* conditional repression strain, detectable amounts of lactate were not observed during the fermentation of TA2821. In addition, the profiles of growth and glycerol consumption between TA2945 and TA2821 were relatively different (Figs. 5a, b, 6a, b). These results suggest that glycerol metabolism toward the central metabolic pathway in the *gapA* conditional repression strain did not depend on the methylglyoxal pathway. It was reported that *E. coli* strains deficient in GAPDH activity did not grow on minimal media containing glucose or glycerol as a sole carbon source. However, the strains were able to grow on media only when containing glycerol together with other substrate such as malate or succinate [41, 42]. These findings imply that the *E. coli* GAPDH deficient strain uses a distinct route for glycerol metabolism. Extensive metabolic flux analyses of the GAPDH deficient strain have never been performed. Therefore the details of the pathway are still unclear.

### Improvement of 3-HP production by *yqhD* deletion

**Fig. 7:**
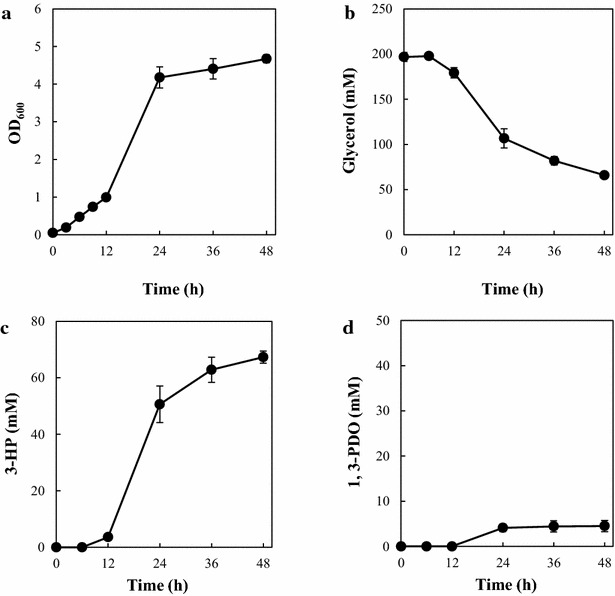
3-HP production by a *gapA* conditional repression strain with deleted *yqhD* (TA3130). **a** Growth curve (OD_600_). **b** Time course of glycerol consumption. **c** Time course of 3-HP production. **d** Time course of 1,3-PDO production. *Error bars* represent the standard deviation (n = 3)

TA2821_9 h (*gapA* conditional repression strain) produced higher concentrations of 1,3-PDO (18.9 ± 3.1 mM) as a major byproduct (Fig. 6d), indicating that endogenous *yqhD* decreased the metabolic flux from 3-HPA to 3-HP in the TA2821. To elevate the 3-HP production and prevent 1,3-PDO production, TA3130, an endogenous *yqhD* deletion strain from TA2821, was constructed (Figs. 1, 2; Table 1). The growth and glycerol consumption of TA3130 were comparable those of wild type strain (TA2463) (Fig. 7a, b). As shown in Fig. 7c, TA3130_9 h produced 67.3 ± 2.1 mM of 3-HP with a yield of 51.5 ± 3.2 % at 48 h, which were about 80 and 94 % higher, respectively, than those of TA2463. The maximum theoretical yield of 3-HP from glycerol under aerobic condition was estimated as 97 % [23]. Thus, the 3-HP yield from glycerol obtained with TA3130 was 53.1 % of the theoretical yield and was comparable to that of our previous report in which the highest 3-HP yield from glycerol (54.1 %) ever reported was achieved using a dual synthetic pathway in flask scale batch fermentation [24].

The titer of 1,3-PDO produced by TA3130_9 h (4.51 ± 1.2 mM) was significantly lower compared to that for TA2821_9 h (18.9 ± 3.1 mM) (Figs. 6d, 7d). The deletion of *yqhD* in the *E. coli* strain producing 3-HP led to reduced 1,3-PDO production. However small amounts of 1,3-PDO was still produced, suggesting that there are other enzymes that can convert 3-HPA to 1,3-PDO in *E. coli* [22, 33]. Additionally, the sum of the concentrations of 3-HP and 1,3-PDO produced by TA3130 (approximately 71.8 mM) was similar to that of TA2821_9 h (approximately 73.1 mM). These results reveal that deletion of *yqhD* did not affect the metabolic flux from glycerol to 3-HPA but decreased the metabolic flux from 3-HPA to 1,3-PDO.

### Measurement of *gapA* activity and intracellular metabolites during fermentation

Previously, we examined whether MTS effectively work as an off switch or not by measuring the enzyme activity that was conditionally repressed and the concentrations of intracellular metabolites [15]. The GAPDH activity in TA2463 (wild type strain), TA3130 (*gapA* conditional repression strain with *yqhD* deletion) without IPTG, and TA3130_9 h were measured at 9 and 24 h. The activities of GAPDH activity in TA3130 without IPTG induction and TA3130_9 h were more than twofold higher (0.655 ± 0.013 and 0.676 ± 0.018 U/mg) than the activity achieved with TA2463 (0.249 ± 0.0037 U/mg) at 9 h, which is likely due to the expression of *gapA* from the medium copy number plasmid in TA3130 and from chromosome in TA2463 (Table 2). The activity of TA2463 at 24 h decreased to half the activity observed at 9 h (0.117 ± 0.012 U/mg), while uninduced TA3130 maintained its activity during the 24 h period (0.576 ± 0.029 U/mg) (Table 2). The difference could be caused by using a native promoter in TA2463 chromosome or the artificial inducible promoter, PltetO_1_ on the plasmid for *gapA* expression even though it has degradation tag at C-terminus. The *gapA* activity of TA3130_9 h at 24 h was 0.0483 ± 0.0031 U/mg, which was 92 and 59 % lower than that for uninduced TA3130 and TA2463, respectively, at 24 h (Table 2). The conditional repression of *gapA* did not significantly reduce the activity as observed in our previous reports of *gltA* conditional repression where the activity decreased by 7 % of the wild type strain [15]. When GAPDH activity is strictly repressed after addition of IPTG, 3-HP titer and yield could be further elevated.

**Table 2:** GAPDH activities during fermentation

Strains	9 h	24 h
TA2463	0.249 ± 0.0037	0.117 ± 0.012
TA3130	0.655 ± 0.013	0.576 ± 0.029
TA3130_9 h	0.676 ± 0.018	0.0483 ± 0.0031

**Fig. 8:**
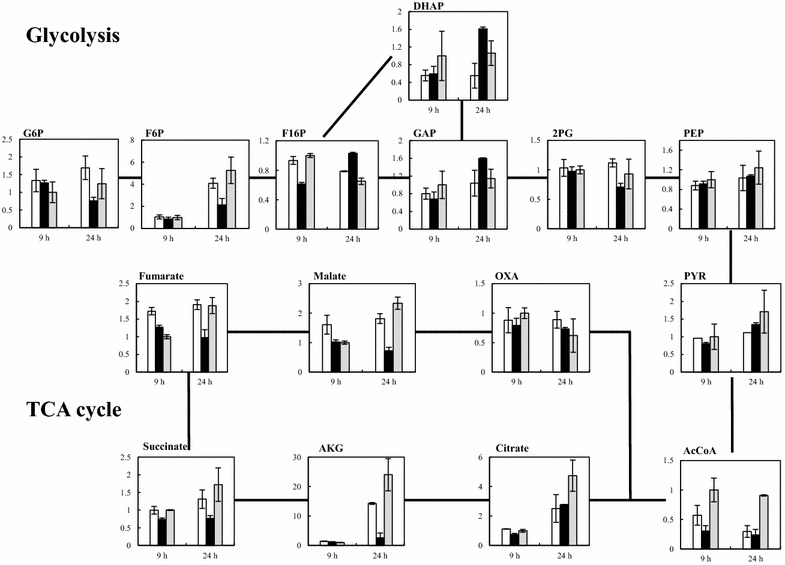
The levels of intracellular metabolites measured by LC–MS analysis. Relative levels of intracellular metabolites in central metabolic pathways were measured by LC–MS. The levels of TA2463 at 9 h were set to 1. Each graph shows intracellular metabolite levels of TA3130, TA3130_9 h, and TA2463. The *horizontal axes* indicate the time of sampling and the *longitudinal axes* indicate the relative levels of intracellular metabolite concentration. *Error bars* represent the standard deviation (n = 3). *White bars* TA3130 without IPTG, *Black bars* TA3130_9 h, *Gray bars* TA2463. Metabolite abbreviations: *G6P* glucose 6-phosphate, *F6P* fructose 6-phosphate, *F16P* fructose 1,6-bisphosphate, *DHAP* dihydroxyacetone phosphate, *GAP* glyceraldehyde 3-phosphate, *2PG* 2-phosphoglycerate, *PEP* phosphoenolpyruvate, *PYR* pyruvate, *AcCoA* acetyl-CoA, *AKG* α-ketoglutaric acid, *OXA* oxaloacetate

LC–MS analysis was performed to investigate the effect of *gapA* conditional repression on intracellular metabolites using cells from TA2463, TA3130 without IPTG, and TA3130_9 h at 9 and 24 h. As shown in Fig. 8, the levels of most of the intracellular metabolites that relate to glycolysis and the TCA cycle were comparable among the three strains at 9 h. However, higher levels of DHAP and GAP were accumulated in TA3130_9 h at 24 h. The level of DHAP in TA3130_9 h was approximately threefold and 1.5-fold higher and of GAP was approximately 1.4-fold and 1.5-fold higher, respectively, than that of TA2463 and TA3130 without IPTG. This probably resulted from the conditional repression of *gapA*. Furthermore, TA3130_9 h had lower levels of several glycolysis and TCA cycle metabolites, such as G6P, F6P, α-ketoglutarate, succinate, fumarate, and malate. These low levels of metabolites indicate the reducing metabolic flux toward central carbon metabolism, and this reduction was most likely to allow for a higher 3-HP titer and yield from TA3110_9 h. Taking into account the results of measurement of GAPDH activity and the concentrations of intracellular metabolites, MTS sufficiently functioned as the off switch of *gapA*. Thus, we demonstrate the state of intracellular metabolites before and after conditional repression of *gapA* in the *E. coliyqhD* deletion mutant. However, details regarding metabolic flux during fermentation are poorly understood. ^13^C-metabolic flux analysis that estimates the intracellular metabolic flux can provide useful information of additional genetic modifications required for further increases in 3-HP production [43].

Recently, Jung et al. demonstrated that deletion of *glpR* encoding a regulation factor repressing the genes for glycerol utilization increased the glycerol utilization rate as well as the 3-HP titer [23]. Chu et al. and Honjo et al. improved the conversion rate of 3-HPA to 3-HP using an aldehyde dehydrogenase mutant or via the introduction of an additional metabolic pathway [24, 44]. The conditional repression of *gapA* combined with these findings provides further improvement of 3-HP productivity. Such resultant strains will be applied to various cultivation conditions including fed-batch culture that could gave knowledge for the eventual commercialization of 3-HP production by *E. coli* with MTS from glycerol.

## Conclusion

Here, we performed conditional repression of genes related to glycerol metabolism (*glpK*, *tipA* and *gapA*) for increasing 3-HP production from glycerol by *E. coli*. The conditional repression of *glpK* and *tpiA* did not improve 3-HP production, indicating that the conditional repression of every node on the routes of carbon utilization does not completely contribute to enhanced productivity of the final products. In contrast, the conditional repression of *gapA* with MTS, accompanied by deletion of *yqhD*, significantly increased 3-HP production from glycerol. The 3-HP titer and yield achieved by the resultant strain TA3130 were 67.3 ± 2.1 mM and 51.5 ± 3.2 %, respectively, which were 80 and 94 % greater, respectively, than those for TA2463, the wild type strain. To our knowledge, this is the first report demonstrating the effectiveness of conditional repression by using the MTS on 3-HP production from glycerol. Taking into account our previous report, which showed increased isopropanol production from glucose by conditional repression of *gltA*, this strategy can be applied to various kinds of bio-production from various substrates.

## Methods

### Chemicals and reagents

Chemicals were purchased from Sigma-Aldrich (St. Louis, MO, USA), Wako Pure Chemical Industries, Ltd. (Osaka, Japan), and MP Biomedicals (Solon, OH, USA), and all restriction enzymes were purchased from New England Biolabs (Ipswich, MA, USA), unless otherwise noted.

### Media and growth conditions

For plasmid preparation, *E. coli* strains were cultured in 3 mL Luria–Bertani (LB) medium in test tubes incubated at 37 °C in a rotary shaker (250 rpm). The media and growth conditions for flask cultures were adopted from a previous study, with slight modifications [24]. *E. coli* strains were grown in M9 minimal media containing 200 mM glycerol, 0.05 % (w/v) yeast extract, and 10 ppm thiamin hydrochloride. Preculture was performed at 37 °C in a rotary shaker (250 rpm), and the main culture was incubated at 37 °C in a rotary shaker (150 rpm) in the dark. Overnight precultures were diluted to 300 mL in a baffled flask containing 25 mL fresh M9 media with initial optical density at 600 nm (OD_600_) of 0.05 as the main culture. IPTG (0.1 mM) and cyanocobalamin (2 μM) were added at the indicated time points (0, 3, 6, or 9 h). All media were supplemented with appropriate antibiotics [kanamycin (50 μg/mL), spectinomycin (100 μg/mL), or chloramphenicol (40 μg/mL)].

### Plasmid and strain construction

The plasmids and strains used in this study are listed in Table 1. All plasmids were prepared in *E. coli* XL1-blue (Agilent Technologies, Santa Clara, CA, USA). PCR analyses were performed using KOD plus NEO (Toyobo Co., Ltd., Osaka, Japan). To insert a DNA fragment containing the tryptophan terminator Ttrp flanked by an IPTG inducible promoter PllacO1 into the pTA867 vector [24] at the BamHI site, a fragment was generated by primer extension using the following primers: T1856 (5′ GCCAT CGGAT CCAGC CCGCC TAATG AGCGG GCTTT TTTTT TCTAG AAATT GTGAG CGGAT AACAA TTGAC ATTG 3′) and T2008 (5′ GCCAT CGGAT CCGGT CAGTG CGTCC TGCTG ATGTG CTCAG TATCT TGTTA TCCGC TCACA ATGTC AATTG TTATC CGCTC ACA 3′). The resultant fragment was digested with BamHI and cloned into the pTA867 vector, resulting in a plasmid designated as pTA1196. The kanamycin-resistance gene of the pTA695 plasmid [15] was replaced with spectinomycin. A fragment encoding the spectinomycin-resistance gene was digested with AvrII and SacI from PZS4Int-laci/tetR [25] and was ligated into the pTA695 vector, which did not contain an antimicrobial-resistance gene, resulting in a plasmid that was designated as pTA1065. *glpK* was amplified from the *E. coli* BW25113 genome by PCR using the following phosphorylated primers T1569: 5′ ATGAC TGAAA AAAAA TATAT CGTTG CGC 3′) and T1702 (5′ TTAAG CTGCT AAAGC GTAGT TTTCG TCGTT TGCTG CTTCG TCGTG TTCTT CCCAC GC 3′); the LAA tag was added at the C-terminus for rapid degradation [26, 27]. The resultant fragment was ligated with a fragment generated from pTA1065 by PCR performed using the primers T2083 (5′ CATGG TACGC GTGCT AGAGG CATC 3′) and T2084 (5′ GGATC CTTTC TCCTC TTTAA TGAAT TCGG 3′), thus creating a pTA1277 plasmid. *gapA* was amplified from the BW25113 genome using the primers T1551 (5′ GCCAT CGGAT CCATG ACTAT CAAAG TAGGT ATCAA CGGTT TTG 3′) and T1704 (5′ GCCAT CACGC GTTTA AGCTG CTAAA GCGTA GTTTT CGTCG TTTGC TGCTT TGGAG ATGTG AGCGA TCAGG TC 3′) for PCR; an LAA tag at the C-terminus. The resultant fragment was digested with BamHI and MluI, and cloned into pTA695, resulting in a plasmid designated as pTA958. The kanamycin-resistance gene of pTA958 was replaced with spectinomycin, similar to the pTA1065 plasmid, resulting in a plasmid designated as pTA1335. To exchange the origin of pTA1065, the plasmid was digested with AvrII and SpeI, and a pTA1065 fragment without origin was gel purified. The resultant fragment was ligated with a pSC101* origin generated from pZS*24MCS [25] by digestion with AvrII and SpeI, resulting in a plasmid designated as pTA1296. *tpiA* was amplified from the BW25113 genome by PCR using the primers T2302 (5′ AAAAA GGATC CATGC GACAT CCTTT AGTGA TGGG 3′) and T2304 (5′ AAAAA GGATC CTTAA GCTGC TAAAG CGTAG TTTTC GTCGT TTGCT GCAGC CTGTT TAGCC GCTTC TG 3′); an LAA tag was added at the C-terminus. The resultant fragment was digested with BamHI and introduced into the BamHI site of pTA1296, creating a plasmid designated as pTA1383. PllacO1-*tpiA*.LAA was amplified by PCR performed using the primers T2404 (5′ AAAAA AGCTT AGCCC GCCTA ATGAG CG 3′) and T2405 (5′ AAAAC CTAGG TCTAG GGCGG CGGA 3′) and digested with AvrII and HindIII. The fragment was cloned into pTA1065 at the AvrII-HindIII site, resulting in a plasmid designated as pTA1393.

All *E. coli* strains used for 3-HP production were based on BW25113. The *gapA* gene was deleted by Wanner method ([29], Nakahigashi unpublished data). The genes *glpK*, *tpiA*, and *yqhD* were inactivated by P1 transduction based on TA1015 or TA2814. TA2793 was constructed by P1 transduction using JW0336 based on TA2732 in which *gapA* was supplemented with pTA1335 [28]. After P1 transduction, the residual kanamycin marker was removed using pCP20 in all strains [29].

### Analytical methods

All analytical methods such as measurement of cell density, extracellular metabolites, glycerol, and intracellular metabolites were performed as previously described [15, 24].

### Glyceraldehyde-3-phosphate dehydrogenase activity

Glyceraldehyde-3-phosphate dehydrogenase (GAPDH) activity was measured as previously reported, with slight modifications [30, 31].* E. coli* strains, TA2463 and TA3130 were cultured in M9 medium as described in section media and growth. Cells were harvested by centrifugation, washed, and resuspended in wash buffer. Crude extracts were prepared by sonication on ice, and supernatants were collected after centrifugation for experiments. The protein concentration of the crude extracts was determined by measuring the absorbance at 280 nm using a NanoDrop 2000 spectrophotometer (Thermo Fisher Scientific, Waltham, USA). The activity was measured by monitoring the increase in absorbance at 340 nm at 25 °C. Crude extracts were added to a reaction mixture containing 200 mM tricine buffer (pH 8.5), 30 mM 2-mercaptoethanol, 10 mM NAD, and 10 mM glyceraldehyde-3-phosphate. Measurement began when 100 mM inorganic phosphate was added. The unit of enzyme activity is defined as the formation of 1 μmol of NADPH per minute.

### Measurement of intracellular metabolites

TA2463 and TA3130 were cultured in M9 medium as described in section media and growth conditions. IPTG (0.1 mM) was added after 9 h of culture for TA3130. Intracellular metabolite extraction and subsequent LC–MS/MS analysis were performed as previously described [15].

## References

[1] Atsumi S, Hanai T, Liao JC (2008). Non-fermentative pathways for synthesis of branched-chain higher alcohols as biofuels. Nature.

[2] Atsumi S, Liao JC (2008). Directed evolution of *Methanococcus jannaschii* citramalate synthase for biosynthesis of 1-propanol and 1-butanol by *Escherichia coli*. Appl Environ Microbiol.

[3] Hanai T, Atsumi S, Liao JC (2007). Engineered synthetic pathway for isopropanol production in *Escherichia coli*. Appl Environ Microbiol.

[4] Borodina I, Kildegaard KR, Jensen NB, Blicher TH, Maury J, Sherstyk S, Schneider K, Lamosa P, Herrgard MJ, Rosenstand I (2015). Establishing a synthetic pathway for high-level production of 3-hydroxypropionic acid in *Saccharomyces cerevisiae* via beta-alanine. Metab Eng.

[5] McKenna R, Nielsen DR (2011). Styrene biosynthesis from glucose by engineered *E. coli*. Metab Eng.

[6] Verhoef S, Ruijssenaars HJ, de Bont JA, Wery J (2007). Bioproduction of p-hydroxybenzoate from renewable feedstock by solvent-tolerant *Pseudomonas putida* S12. J Biotechnol.

[7] Chen X, Zhou L, Tian K, Kumar A, Singh S, Prior BA, Wang Z (2013). Metabolic engineering of *Escherichia coli*: a sustainable industrial platform for bio-based chemical production. Biotechnol Adv.

[8] Clomburg JM, Gonzalez R (2011). Metabolic engineering of *Escherichia coli* for the production of 1,2-propanediol from glycerol. Biotechnol Bioeng.

[9] Kim J, Copley SD (2007). Why metabolic enzymes are essential or nonessential for growth of *Escherichia coli* K12 on glucose. Biochemistry.

[10] Callura JM, Cantor CR, Collins JJ (2012). Genetic switchboard for synthetic biology applications. Proc Natl Acad Sci USA.

[11] Cho HS, Seo SW, Kim YM, Jung GY, Park JM (2012). Engineering glyceraldehyde-3-phosphate dehydrogenase for switching control of glycolysis in *Escherichia coli*. Biotechnol Bioeng.

[12] Lu J, Tang J, Liu Y, Zhu X, Zhang T, Zhang X (2012). Combinatorial modulation of *galP* and *glk* gene expression for improved alternative glucose utilization. Appl Microbiol Biotechnol.

[13] Solomon KV, Sanders TM, Prather KL (2012). A dynamic metabolite valve for the control of central carbon metabolism. Metab Eng.

[14] Gardner TS, Cantor CR, Collins JJ (2000). Construction of a genetic toggle switch in *Escherichia coli*. Nature.

[15] Soma Y, Tsuruno K, Wada M, Yokota A, Hanai T (2014). Metabolic flux redirection from a central metabolic pathway toward a synthetic pathway using a metabolic toggle switch. Metab Eng.

[16] Valdehuesa KN, Liu H, Nisola GM, Chung WJ, Lee SH, Park SJ (2013). Recent advances in the metabolic engineering of microorganisms for the production of 3-hydroxypropionic acid as C3 platform chemical. Appl Microbiol Biotechnol.

[17] Bozell JJ, Petersen GR (2010). Technology development for the production of biobased products from biorefinery carbohydrates -’s ‘‘Top 10’’ revisited. Green Chem.

[18] Hu S, Luo X, Wan C, Li Y (2012). Characterization of crude glycerol from biodiesel plants. J Agric Food Chem.

[19] Yazdani SS, Gonzalez R (2007). Anaerobic fermentation of glycerol: a path to economic viability for the biofuels industry. Curr Opin Biotechnol.

[20] Mohan Raj S, Rathnasingh C, Jung WC, Park S (2009). Effect of process parameters on 3-hydroxypropionic acid production from glycerol using a recombinant *Escherichia coli*. Appl Microbiol Biotechnol.

[21] Rathnasingh C, Raj SM, Jo JE, Park S (2009). Development and evaluation of efficient recombinant *Escherichia coli* strains for the production of 3-hydroxypropionic acid from glycerol. Biotechnol Bioeng.

[22] Tokuyama K, Ohno S, Yoshikawa K, Hirasawa T, Tanaka S, Furusawa C, Shimizu H (2014). Increased 3-hydroxypropionic acid production from glycerol, by modification of central metabolism in *Escherichia coli*. Microb Cell Fact.

[23] Jung WS, Kang JH, Chu HS, Choi IS, Cho KM (2014). Elevated production of 3-hydroxypropionic acid by metabolic engineering of the glycerol metabolism in *Escherichia coli*. Metab Eng.

[24] Honjo H, Tsuruno K, Tatsuke T, Sato M, Hanai T (2015). Dual synthetic pathway for 3-hydroxypropionic acid production in engineered *Escherichia coli*. J Biosci Bioeng.

[25] Lutz R, Bujard H (1997). Independent and tight regulation of transcriptional units in *Escherichia coli* via the LacR/O, the TetR/O and AraC/I1-I2 regulatory elements. Nucleic Acids Res.

[26] Keiler KC, Waller PR, Sauer RT (1996). Role of a peptide tagging system in degradation of proteins synthesized from damaged messenger RNA. Science.

[27] Prindle A, Samayoa P, Razinkov I, Danino T, Tsimring LS, Hasty J (2012). A sensing array of radically coupled genetic ‘biopixels’. Nature.

[28] Baba T, Ara T, Hasegawa M, Takai Y, Okumura Y, Baba M, Datsenko KA, Tomita M, Wanner BL, Mori H (2006). Construction of *Escherichia coli* K-12 in-frame, single-gene knockout mutants: the Keio collection. Mol Syst Biol.

[29] Datsenko KA, Wanner BL (2000). One-step inactivation of chromosomal genes in *Escherichia coli* K-12 using PCR products. Proc Natl Acad Sci USA.

[30] Martinez I, Zhu J, Lin H, Bennett GN, San KY (2008). Replacing *Escherichia coli* NAD-dependent glyceraldehyde 3-phosphate dehydrogenase (GAPDH) with a NADP-dependent enzyme from *Clostridium acetobutylicum* facilitates NADPH dependent pathways. Metab Eng.

[31] Iddar A, Valverde F, Serrano A, Soukri A (2002). Expression, purification, and characterization of recombinant nonphosphorylating NADP-dependent glyceraldehyde-3-phosphate dehydrogenase from *Clostridium acetobutylicum*. Protein Expr Purif.

[32] Jarboe LR (2011). YqhD: a broad-substrate range aldehyde reductase with various applications in production of biorenewable fuels and chemicals. Appl Microbiol Biotechnol.

[33] Kim K, Kim SK, Park YC, Seo JH (2014). Enhanced production of 3-hydroxypropionic acid from glycerol by modulation of glycerol metabolism in recombinant *Escherichia coli*. Bioresour Technol.

[34] Kwak S, Park YC, Seo JH (2013). Biosynthesis of 3-hydroxypropionic acid from glycerol in recombinant *Escherichia coli* expressing *Lactobacillus brevis dhaB* and *dhaR* gene clusters and *E. coli* K-12 *aldH*. Bioresour Technol.

[35] Martinez-Gomez K, Flores N, Castaneda HM, Martinez-Batallar G, Hernandez-Chavez G, Ramirez OT, Gosset G, Encarnacion S, Bolivar F (2012). New insights into *Escherichia coli* metabolism: carbon scavenging, acetate metabolism and carbon recycling responses during growth on glycerol. Microb Cell Fact.

[36] Oh MK, Liao JC (2000). Gene expression profiling by DNA microarrays and metabolic fluxes in *Escherichia coli*. Biotechnol Prog.

[37] Peng L, Shimizu K (2003). Global metabolic regulation analysis for *Escherichia coli* K12 based on protein expression by 2-dimensional electrophoresis and enzyme activity measurement. Appl Microbiol Biotechnol.

[38] Schuetz R, Kuepfer L, Sauer U (2007). Systematic evaluation of objective functions for predicting intracellular fluxes in *Escherichia coli*. Mol Syst Biol.

[39] Hopper DJ, Cooper RA (1971). The regulation of *Escherichia coli* methylglyoxal synthase; a new control site in glycolysis?. FEBS Lett.

[40] Noda S, Takezawa Y, Mizutani T, Asakura T, Nishiumi E, Onoe K, Wada M, Tomita F, Matsushita K, Yokota A (2006). Alterations of cellular physiology in *Escherichia coli* in response to oxidative phosphorylation impaired by defective F1-ATPase. J Bacteriol.

[41] Irani MH, Maitra PK (1977). Properties of *Escherichia coli* mutants deficient in enzymes of glycolysis. J Bacteriol.

[42] Hillman JD, Fraenkel DG (1975). Glyceraldehyde 3-phosphate dehydrogenase mutants of *Escherichia coli*. J Bacteriol.

[43] Toya Y, Ishii N, Nakahigashi K, Hirasawa T, Soga T, Tomita M, Shimizu K (2010). ^13^C-metabolic flux analysis for batch culture of *Escherichia coli* and its Pyk and Pgi gene knockout mutants based on mass isotopomer distribution of intracellular metabolites. Biotechnol Prog.

[44] Chu HS, Kim YS, Lee CM, Lee JH, Jung WS, Ahn JH, Song SH, Choi IS, Cho KM (2015). Metabolic engineering of 3-hydroxypropionic acid biosynthesis in *Escherichia coli*. Biotechnol Bioeng.

